# Assessing the impact of missing genotype data in rare variant association analysis

**DOI:** 10.1186/1753-6561-5-S9-S107

**Published:** 2011-11-29

**Authors:** Reedik Mägi, Ashish Kumar, Andrew P Morris

**Affiliations:** 1 Wellcome Trust Centre for Human Genetics, University of Oxford, Oxford, UK; 2 Swiss Tropical and Public Health Institute, University of Basel, Basel, Switzerland

## Abstract

Human genome resequencing technologies are becoming ever more affordable and provide a valuable source of data about rare genetic variants in the human genome. Such rare variation may play an important role in explaining the missing heritability of complex human traits. We implement an existing method for analyzing rare variants by testing for association with the mutational load across genes. In this study, we make use of simulated data from the Genetic Analysis Workshop 17 to assess the power of this approach to detect association with simulated quantitative and dichotomous phenotypes and to evaluate the impact of missing genotypes on the power of the analysis. According to our results, the mutational load based rare variant analysis method is relatively robust to call-rate and is adequately powered for genome-wide association analysis.

## Background

The success of genome-wide association studies (GWAS) to identify novel loci that contribute to complex human traits has been well publicized [1]. However, despite these successes, much of the genetic component of these traits remains unexplained. Most genotyping products that are used in GWAS have been designed to capture common human genetic variation [2], and with ever increasing sample sizes and meta-analysis, we might expect to identify associations with common variants with ever decreasing effect size. However, it seems unlikely that the common disease/common variant model will entirely explain the missing heritability of complex traits. One widely unexplored paradigm that may contribute to this unexplained genetic component is a model of multiple rare causal variants, defined here as those having a minor allele frequency (MAF) less than 5%, each with modest effect but residing within the same gene. Such an association has recently been identified between rare variants in the *IFIH1* gene and type 1 diabetes [3].

Until recently, the availability of data appropriate for rare variant association analysis has been extremely limited. However, with improvements in the efficiency of deep resequencing technologies, discovery and analysis of rare variants is becoming increasingly cost-effective and financially feasible in large disease- or population-based cohorts at the level of specific genes or even exome-wide. Furthermore, large-scale whole-genome resequencing efforts, such as the 1000 Genomes Project [4], continue to make their data available to the research community. These resources are likely to provide near complete catalogs of low-frequency genetic variation and of many other rarer variants in a variety of populations across ethnic groups. These data can provide deep and high-density reference panels, potentially allowing for imputation of rare variants that are not typically directly assayed or otherwise captured by genotyping products in GWAS [5].

One common approach to the joint analysis of rare variants within the same gene is to focus on their mutational load, searching for accumulations of minor alleles across individuals with the same or similar phenotype [6,7]. Simulation studies have demonstrated that such an approach has much greater power to detect rare variant associations than traditional single-SNP analyses [6,7]. However, these studies typically assume no genotyping and/or sequencing failures, which, particularly for rare variants, may affect the results of their downstream analysis. In this study, we undertake simulations to assess the effects of missing genotype data on rare variant association analysis. We use the simulated data from Genetic Analysis Workshop 17 (GAW17), which includes genotypes at exonic rare variants within a subset of genes across the genome, generated from the 1000 Genomes Project [4]. We make use of a simple model of random missing genotypes and evaluate the effect of failure rate on the power of mutational load rare variant association with quantitative and dichotomous traits. Analysis of pilot data from the 1000 Genomes Project shows that the mutant (rare) allele is more difficult to call than the reference (common) allele. To mimic this allele-specific failure rate, we have incorporated into our analysis a more complex model of missing data in which the call rate is determined by the underlying genotype.

## Methods

### Rare variant mutational load analysis

Consider a sample of unrelated individuals typed for rare variants within the same gene. Let *n_i_* denote the number of rare variants for which the *i*th individual has been successfully genotyped, and let *r_i_* denote the number of these variants for which the individual carries at least one copy of the minor allele. We can model the phenotype *y_i_* of the *i*th individual in a generalized linear modeling (GLM) framework, given by:(1)

where *g* is the link function, **x***_i_* denotes a vector of covariate measurements for the *i*th individual with corresponding regression coefficients **β**, and parameter *λ* is the expected increase in the phenotype for an individual carrying a full complement of minor alleles at rare variants compared to an individual carrying none. Thus we construct a likelihood ratio test of association of the mutational load of rare variants with disease by comparing the maximized likelihoods of two models by means of analysis of deviance: (1) the null model where *λ* = 0 and (2) the alternative model where *λ* is unconstrained. The contribution of the *i*th individual to the likelihood is weighted by *n_i_* to allow for differential call rates between samples.

The described method has been implemented using the GRANVIL software, which is freely available for download from http://www.well.ox.ac.uk/GRANVIL. The software can be applied to quantitative traits and dichotomous phenotypes and can adjust for potential confounders as covariates. Users must provide a list of genes, with start and stop positions, together with a map file for variant locations.

### GAW17 data

The data provided by GAW17 contain genotype data for 697 individuals from the 1000 Genomes Project [4]. Individuals were chosen from different populations with European, Asian, and African origin. Overall, 24,487 variants from 3,205 gene regions were provided with MAFs in the range 0.07–16.6%. Three normally distributed quantitative traits and a dichotomous disease phenotype were simulated for each individual on the basis of their genotype data. Q1 and Q2 phenotypes were determined by genotypes in 9 and 13 genes, respectively. Q4 was not determined by any variants among the genes provided. Disease liability was generated using a function of Q1, Q2, and Q4 phenotypes in addition to variants in a further 15 genes. Two hundred replicates of data were simulated, each on the basis of the same underlying genotypes and each stored in a separate phenotype file. Full details of the GAW17 data and simulation approach used to generate the phenotype data are reported elsewhere [8].

### Simulation study

We make use of the simulated GAW17 data to investigate the type I error rate and power of GRANVIL to detect association with quantitative traits Q1, Q2, and Q4 and the dichotomous disease (CC) phenotype. We consider rare variants to have MAF < 5%. GRANVIL gives equal weight to all these rare variants in the gene, irrespective of their potential functional role. We therefore performed two analyses of each replicate of phenotype data: (1) including all rare variants, irrespective of function; and (2) restricting rare variants to those that are nonsynonymous. We used GRANVIL to test for association with the mutational load in each gene containing at least two rare variants. Phenotype data for individual NA07347 was excluded because of extreme deviation from the mean in most replicates [9]. For each analysis, all phenotypes were adjusted for sex, age, and smoking status. GAW17 individuals were ascertained from three major ethnic groups: (a) European origin (European Americans [CEPH], Tuscan); (b) Asian descent (Denver Chinese, Han Chinese, Japanese); and (c) African ancestry (Yoruba and Luhya). Population stratification analysis revealed separate clusters for these major ethnic groups (data not shown). To avoid problems arising from stratification, we thus performed GRANVIL analyses for each ethnic group separately and combined the results for each gene using inverse-variance fixed-effects meta-analysis of the parameter *λ*, implemented in the GWAMA software [10].

To assess the effect of genotype call rate on type I error rates and power, we randomly removed rare variant genotypes from individuals to simulate missing data. We considered a range of missing data rates: 0.1%, 0.5%, 1%, 5%, and 10% of all available genotypes. To take account of the possibility of allele-specific failure rates, we also considered a more complex model in which heterozygous and rare homozygous genotypes were more difficult to call. Specifically, we randomly removed 1% of common homozygous genotypes, 5% of heterozygous genotypes, and 10% of rare homozygous genotypes. For each model of missing genotype data, we generated 1,000 replicates of data, each from a randomly selected phenotype file from GAW17.

The power (type I error rate) was assessed at a nominal Bonferroni-corrected threshold of *p* ≤ 3.86 × 10^−5^ (0.05/1,297 genes having at least two rare variants). We assessed power by considering all genes known to be causal for the respective phenotype and calculated type I error rate by considering all noncausal genes [8].

## Results

Analysis of all rare variants and analysis of only nonsynonymous rare variants generated qualitatively similar results. We thus present here the results for the analysis of nonsynonymous variants.

**Figure 1:**
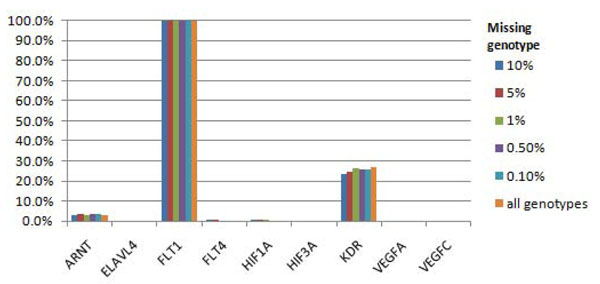
**Power to detect associations for Q1 phenotype using nonsynonymous markers.** All gene regions affecting Q1 phenotype are presented.

Despite the simulated rate of missing genotype data, we were able to detect the association of the causal *FLT1* gene with Q1 in 100% of the runs (Figure 1). The causal *KDR* gene was detected in 23.2% of replicates with a 90% random call rate, in 23.8% of replicates for our allele-specific model, and in 26.8% of replicates when there was no missing genotype data. For the rest of the causal genes for Q1, we had low power to detect associations with *ARNT* (up to 3.9% of replicates, depending on the call rate) and *HIF1A* (0.5% of replicates with a 90% random call rate, 0.6% with a 95% random call rate, and just 0.3% with our allele-specific model). Interestingly, the power of *HIF1A* was lower in our models with higher random call rates, although this is likely to reflect stochastic variation in our simulations. The type I error rate for the detection of association with Q1 was higher than expected in several noncausal genes, including *OR2T34*, *OR2T3*, *NOMO1*, and *HLA-B.* The high type I error rates remained, irrespective of the call rate; for example, association with *OR2T34* was detected in 78.4% of replicates for a 90% random call rate and increased to 85.9% of replicates when there were no missing data. Thus these type I errors have not occurred as a result of missing genotypes but because of extended linkage disequilibrium between rare variants across chromosomes.

**Figure 2:**
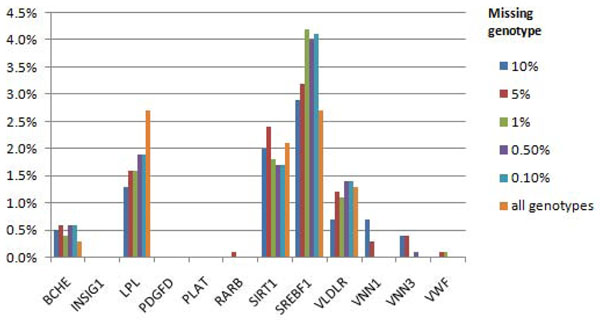
**Power to detect associations for Q2 phenotype using nonsynonymous markers.** All gene regions affecting Q2 phenotype are presented.

**Figure 3:**
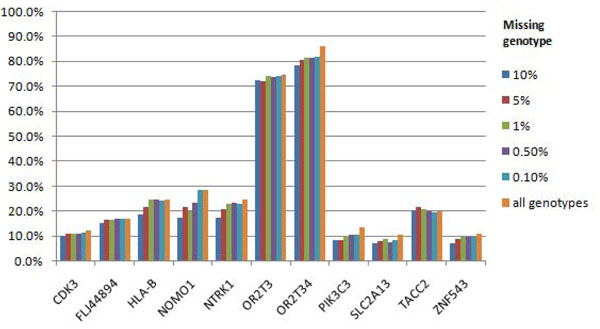
**False-positive associations for Q4 phenotype using nonsynonymous markers**. The ten most associated gene regions are presented.

For Q2, we had power to detect association with several causal genes, namely, *BCHE*, *LPL*, *SIRT1*, *SREBF1*, and *VLDRL*, but only in a small percentage of replicates (up to 4.2% with a 99% call rate) (Figure 2). The type I error rates for Q2 were lower than those for Q1. For Q4, which is not associated with variants in any gene, the false-positive error rate was never higher than 1.1% (Figure 3).

**Figure 4:**
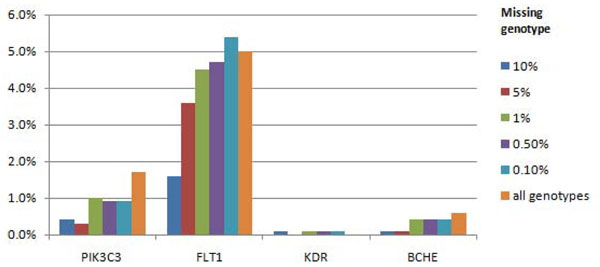
**Power to detect associations for disease status using genes underlying disease liability and the genes affecting Q1 and Q2 phenotypes using nonsynonymous markers.** Only gene loci with power larger than 0 are presented.

For the disease (CC) phenotype, we were able to detect the causal *FLT1* gene locus in 5% of replicates with no missing genotype data, 5.3% of replicates with a 99.9% call rate and the allele-specific model, and only 1.6% of replicates with a 90% random call rate. The second-ranked causal gene was *PIK3C3*, identified in just 1.7% of replicates with no missing genotype data (Figure 4). In addition, the false-positive *OR2T3* and *OR2T34* genes, which showed associations with the Q1 phenotype, showed associations in 1.1% and 1.0%, respectively, of the runs with the full data set accordingly.

## Discussion

One of the key advantages of testing for association of the mutational load within a gene is that we can take account of multiple rare variants simultaneously [7]. Our results demonstrate that we have high power to detect association with rare variants in some of the causal genes for Q1, Q2, and the disease (CC) phenotype. Furthermore, our results suggest that missing genotype data, with call rates as low as 90%, have little effect on power. The mutational load association analysis implemented in GRANVIL weights the contribution of each individual to take account of missing genotypes. Our results suggest that GRANVIL is robust to call rates as low as 90%. There was evidence of increased type I error rates for several noncausal genes, particularly for Q1. However, this reflects long-range linkage disequilibrium between rare variants rather than sensitivity to missing genotype data.

We considered two models of missing genotype data: random failure and an allele-specific model that gives greater probability to uncalled heterozygous and rare homozygous genotypes. Our results were consistent across these two models. This is presumably because for rare variants most of the genotypes are common homozygotes and are thus more robust to call rates determined by the presence of a minor allele.

In this paper, we considered the effect of missing genotype data on the power and type I error rates of a method that tests for association of the mutational load of rare variants within genes. However, sequence and genotyping errors also play an important role in the performance of any association approach for common or rare variants. Analysis of the pilot data from the 1000 Genomes Project suggests greater concordance with HapMap for common homozygous genotypes (more than 99%) than for heterozygous or rare homozygous genotypes (95–98%). The simulated GAW17 data could also be used to assess the effect of a range of sequencing and genotyping error models on the performance of rare variant mutational load analyses.

## Conclusions

The results of our analysis of the simulated GAW17 data suggest that the GRANVIL approach for testing association with the mutational load of rare variants within a gene is relatively robust to missing genotype data, occurring either at random or with differential allele-specific failures. Our power to detect association with causal genes was not dramatically affected by call rate. Similarly, the type I error rate for noncausal genes is relatively unaffected by the rate of missing genotypes but is somewhat inflated by the extent of long-range linkage disequilibrium between noncausal genes.

## Competing interests

The authors declare that there are no competing interests.

## Authors’ contributions

RM participated in the design of the study, performed the statistical analysis and drafted the manuscript. AK participated in the statistical analysis and helped to draft the manuscript. AM conceived of the study, and participated in its design and coordination and helped to draft the manuscript. All authors read and approved the final manuscript.
